# Report of a Case of Premature Labour, Artificially Induced, with Successful Results, at the Seventh Month of Gestation, on Account of Contracted Pelvis

**Published:** 1850-06

**Authors:** 


*Report of a case of premature labour, artificially induced, with successful results, at the seventh month of gestation, on account of contracted pelvis.* By Thomas W. Blatchford, M. D. Read before the Rensselaer county Medical Society, Jan. 7, 1850.

Mrs. M., thirty-one years of age, short and thick-set, of sanguine temperament and good constitution, was married Dec. 25th, 1845. On the 11th of September, 1846, I was sent for to attend her in her first confinement. The membranes had broken early in the morning, and without pain; pains however soon succeeded, and when I first saw her they were regular and quite severe. Upon examination *per vaginam*, the os tincæ was found but little if at all acted upon, and it was not until the second day that the dilatation was sufficient to ascertain that the head presented. After bleeding, and a full dose of opium, dilatation progressed more rapidly, and was completed about the end of the second day; but notwithstanding the pains for the most part had been severe and forcing, with very short intervals of ease, at the close of the third day the head had progressed only through the upper strait. It now became very doubtful whether labor could be terminated without instrumental assistance. The advice of able counsel was now sought. In consultation it was agreed that inasmuch as the head still receded a little upon pressure, the pains still very forcing, the pulse firm and good, and no marked signs of prostration present, we should still leave the case to nature for a few hours longer. At nine o'clock A. M. we met again. The pains had continued unabated. The head was found in about the same position, but it now seemed completely impacted. The scalp was soft and patulous, no fœtal motion discernible upon examination per vaginam. Yet the patient said she distinctly felt life, and the stethescope confirmed the assertion. Her pulse was somewhat quickened, and she had become very restless, and anxious that the child should be taken away immediately with instruments. We attempted the introduction of the forceps, but the pressure between the bones of the head and those of the pelvis was so great, that their introduction became impossible without endangering the soft parts of the mother. The vectis was tried, but with no better success; and the only alternative was the perforator and the blunt hook, which affected delivery after about half an hour's effort. The child had been dead long enough to smell offensive, and the cuticle was detached in several places. It weighed a little over six pounds. In tracing the umbilical cord for the placenta, it was discovered that a bag of water presented. It was that of a second child, and a footling case. The membranes were ruptured, the feet graspcd, and the labor terminated within about five minutes. The child was in a state of asphyxia, and remained so several minutes, but perseverance in the use of the hot spirit bath and artificial respiration, at length restored it to life. It weighed four pounds, soon became vigorous, and grew finely, and is now, 1850, a very healthy girl, between three and four years of age.

In about fourteen months after that time, Mrs. M. whose health had been excellent, found herself at the full term of a second pregnancy. On the 26th of November, 1847, labor commenced. It progressed much after the same manner as before. It was introduced by the rupture of the membranes. The capacity of the pelvis seemed less if possible than at the first confinement. It did not appear deformed at any one particular point, but of contracted dimensions in every diameter. After the first twenty-four hours her pains were almost constant, seldom affording her a five minutes' interval of ease. The tardy dilation of the os tincæ did not seem to interpose any very serious obstacle to the descent of the head, (the head presented,) for it was about as slow after perfect dilatation had taken place as it was before. Bleeding as before was resorted to, but with very little apparent benefit, except in preventing inflammation. Opium she refused to take, in consequence of some unpleasant symptoms which before resulted from its administration.

Nearly seventy hours had now elapsed, and the prospect of delivery without instrumental assistance, seemed as remote as ever. The strength of the patient became exhausted. Her pulse was over a hundred. She insisted that she had not "felt life" for two days, and no signs of life were discoverable either by taxis or stethoscope. The entreaties of both patient and friends became urgent to have me terminate labor as before. At this stage further medical advice was requested. After a careful examination into the case, it was determined to wait no longer, but to use the perforator at once, as offering the only safe course to the mother. The perforator was introduced; the brain and most of the bones of the cranium were extracted, and yet nearly two hours were consumed before labor was terminated. The child weighed nearly eight pounds, and did not exhibit any signs of having been lcng dead. The patient recovered after an unusually short confinement, and felt no other inconvenience than a slight laceration of perineum, which healed entirely in about two weeks.

From these two trials it became very evident that Mrs. M. could never have a l ving child of ordinary size at maturity. We were therefo-e necessarily driven to the conclusion, that this was one of those cases which would justify the induction of premature labor, at a period when the viability of the child might be reasonably calculated upon. Accordingly she was now promised, that should she ever again become pregnant, labor should be brought on at the seventh month, when the child would not probably weigh over four or five pounds, and when its life would not necessarily be endangered. She was further told that her labor would in all probability be very short, terminating after a very few hours' continuance.

During the last summer, it became evident to friends, that Mrs. M. was again pregnant. She was last unwell 5th May. What doubts she may have entertained herself, were all dissipated when she felt life, the last of September. Very soon she sent for me, and reminded me of my promise. As the seventh month approached, she became increasingly anxious about her situation, and desirous to have labor induced, just as soon as we thought it would answer. Her healty had been excellent. She had experienced no other inconvenience from her situation than an obstinate costiveness, and rather an unwielded weight of abdomen. She was unusually large for one at seven months. Her size was thought to be greater than that of most women at nine months.

On Wednesday, 5th of December, 10 o'clock, A. M., being just seven months since she was last unwell, and two and a half since she quickened, every thing being in readiness, with the assistance of Dr. Robbins, half a pint of "tar water" was injected into the womb through a large-sized male catheter, moderately curved, and by means of the syringe of a common self-injecting apparatus. The patient was placed upon her left side, with her knees separated. The fore-finger of my left hand placed upon the posterior lip of the os tincæ, guiding the catheter in its introduction. It passed without the least resistance from two to two and a half inches within the uterus, occasioning not the slightest pain. No fluid escaped from the catheter. The patient then turned upon her back, and was requested to take hold of the catheter herself, and not suffer it to move either backward or forward, which she did. The syringe was then attached to the catheter, and the injection slowly and cautiously passed, to avoid, if possible, the rupture of the membranes. Upon detaching the syringe, a few spoonfuls of fluid escaped through the catheter, tinged with blood, which at first we feared was the liquor amnii. The operation lasted but a few moments.

After remaining about ten minutes in a recumbent posture, she was permitted to get up, which she did, and moved about the house as usual, experiencing no other inconvenience than a constant draining from the vagina, of a small quantity of fluid slightly tinged with blood and tainted with tar, and a sense of weight as if, to use her own expression, "the child had settled down."

Nothing unusual occurred until Friday evening, the seventh, when she was suddenly taken with a chill and rigor, which lasted nearly two hours, accompanied with severe headache. It was succeeded by slight fever. She, however, rested tolerably well during the night, having bathed her feet and taken an active cathartic.

Saturday morning I found her very comfortable, as much so as she had been for weeks, with the exception of a slight draining before mentioned, which, however, she said was not sufficient to require her to wear a napkin. At eleven o'clock, however, and after the operation of the cathartic, she was taken in labor. The pains at first were few and far between, until about one o'clock, P. M., when they became quite violent and frequent. At two o'clock the membranes gave way during a hard pain, and a very large quantity of water was disaharged. The nurse and patient both say, more than two quarts. It diminished her size very much; so much that when I entered tee room, having been sent for in haste, I was saluted with, "Doctor, see here, I have had my baby, and it is all water." The effect of this large evacuation was to give an almost entire relief from pain.

Upon examination *per vaginam*, no impression had apparently been made upon the os tincæ. During the afternoon and evening she continued free from any severe pain, and rested that night quite as well as she did the night previous. She felt occasionally a heavy bearing down sensation, and once in a while an acute pain in her back; occasioned, she said, by the uncommon motion of the child. If I could give her any thing to make that lie still, she was sure she should not have any pain.

By a little after eight o'clock, Sabbath morning, he pains again returned, at first slight and not very frequent, but they soon became very regular, the interval being about five minutes. An examination at this period detected no change upon the os tincæ. The point of the finger could hardly enter it, still the soft parts were not at all heated, and they were now well lubricated. It became evident that she was even now to have a tedious labor, notwithstanding the caution used, It was not till noon that dilatation could be said to have fairly commenced. The pains, though regular, did not assume any very great degree of severity till about five o'clock, when they began to be very forcing. She complained mostly of her back. Dilatation now went on more rapidly, and by eight o'clock the head nould be felt forcing its way through the upper straight. From this time until one o'clock, the pains were very severe, and yet very little progress had apparently been made towards the completion of labor. Dilatation, however, was now perfect. So far so good. But the patient, hitherto firm and resolute, began to manifest signs of restlessness and impatience, and her spirits evidently began to flag. If she could have pain any where besides in her back, she said she could bear it without a complaint. Her cry, and that of some friends, was for me to terminate labor immediately by instruments, or give her something to put her out of misery; she wanted " to die and not to live," &c., &c.

The head was slightly moveable, receding a little after each pain, advancing, however, but little, if any further, during a succeeding effort. I was tempted at this stage to administer the ergot, or to employ the vectis, but the evident viability of the fœtus, the perfect dilatation of the os uteri, the thorough lubrication of the soft parts, and their entire freedom from any undue heat, together with the undiminished energy of the pains, and with all the comforting knowledge that a small child had once passed through the same aperture, encouraged my non-interference. Besides, I must confess, I felt a mighty unwillingness to resort to any aid either medicinal or instrumental, in a seven-month case, or do anything whereby I might endanger the life of the child, so long as the mother's safety did not clearly require it. Under these circumstances, therefore, I determined to leave the case still longer to nature. More than once I regretted the promise I had given her of having an easy time. I was often twitted about it, and asked if "I called that a quick and easy time," &c.

From about half past one oclock, the pains were nearly continuous, and at times exceedingly severe, resembling much those induced by the administration of ergot, and thus they continued increasing in force and severity if possible, until half past two, A. M., (113 hours from the time the tar water was injected,) when she was delivered of a plump and vigorous child, loudly vociferating its own advent. It weighed nearly four pounds. The placenta soon followed. The secretion of milk was established in the usual time, and the child required no lessons of instructions to draw it, taking the breast as promptly and as eagerly as if it had been a nine, instead of a seven months' prouuction. The mother recovered without any unpleasant symptoms whatsoever. The almost necessary "soreness and stiffness" after so much exertion, soon passed off, and in ten days she was up and about the room, and in one fortnight dismissed her nurse, assumed the discharge of her domestic affairs, and has the satisfaction, to use her own expression, "of nursing her own infant, with as fair a prospect of raising it as any other mother enjoys." Her sufferings, it is true, were very great, but in her own estimation even, and in that of those who witnessed both, they were not near as severe as those she had bofore undergone.

To my mind, the five methods of inducing premature labor given by Churchill, in his "system of midwifery,"—and he devotes a whole chapter to the subject, with which every one intending to operate, would do well to make himself familiar,—did not seem to offer the advantages which the simple injection of "tar water" presented. The ergot might endanger the life of the child, about one half being still-born after its employment. To puncturing the membranes, and letting off the waters either suddenly, or little by little, necessarily subjecting the child to great pressure at a period when the tenacity of life is very feeble, there was the same objection. The contracted capacity of the vagina would not in this case permit the "introduction of the hand sufficiently far to datatch the membranes with the finger" without causing excessive pain, or even introduce into the os uteri the spunge of Kluge. An attempt to detatch the membranes for an inch or two within the os, by means of a catheter, seemed almost of a necessity to endanger the integrity of the membranes; and "abdominal frictions and manipulations, and the warm bath," seemed to be remedies entirely too domestic, unscientific and uncertain.

M. Cohen, of Hamburg, the first I believe to propose injections, states that he had been led to try them for the purpose of inducing premature labor, from noticing their power in developing contractions when introduced into the unimpregnated uterus, "and as," says he, "the pregnant uterus is in a condition apt to contract, he thought injections might be efficatiously used, and that without danger, to bring a delivery in those cases where it is necessory the fœtus should be expelled before the full period of pregnancy." He states further that the "he had been in the habit of employing "tar water" for diminishing the excess secretions from the uterine surface," and thus he was led to make use of it for this object as in the case he has detailed.

The reader of M. Cohen's paper, as noticed in several of the leading Medical Periodicals, since 1847, will perceive that I did not repeat the injection after six hours, as M. Cohen recommends. I did not repeat it at all, for the reason that the constant, though slow draining of fluid more or less tinged with blood, led me to believe that the membranes had been ruptured, and notwithstanding all my care to prevent such an occurrence; and I continued of that opinion until informed of the copious and sudden discharge of water after two or three hours severe labor; otherwise, I should have followed M. Cohen's directions to the letter.

NOTE.—March 15, 1850. The child to-day weighs 12½ [illegible] en has suffered from "*sprue, red gum* and *jaundice.*" The mother's hea'thi [illegible] t, and the secretion of milk abundant.—*Transactions New York State Medical Society*'

